# 2- to 20-year myelomeningocele follow-up outcomes from a referral center in Southern Iran: the Shiraz experience

**DOI:** 10.1186/s40001-024-01667-0

**Published:** 2024-03-25

**Authors:** Sina Zoghi, Mohammad Amin Mosayebi, Maryam Feili, Hossein Eskandari, Hadis Jalalinezhad, Mohammad Sadegh Masoudi, Reza Taheri

**Affiliations:** 1 Department of Neurosurgery, Shiraz University of Medical Sciences, Shiraz, Iran; 2 Student Research Committee, Shiraz University of Medical Sciences, Shiraz, Iran; 3 Student Research Committee, Fasa University of Medical Sciences, Fasa, Iran; 4 Shiraz Neuroscience Research Center, Shiraz University of Medical Sciences, Shiraz, Iran; 5 School of Medicine, Fasa University of Medical Sciences, Fasa, Iran

**Keywords:** Meningomyelocele, Outcome, Incontinence, Follow-up, Education

## Abstract

**Background:**

The current convention for treatment of children with myelomeningocele (MMC) is timely surgical intervention combined with long-term follow-up by a multidisciplinary specialized team. This study aims to investigate the outcomes of MMC patients treated at Namazi Hospital.

**Methods:**

All children presenting to Namazi Hospital with myelomeningocele between May 2001 and August 2020 were eligible for this study. For those with a documented telephone number, follow-up phone surveys with the patient’s caregivers, on top of the review of the medical documents were carried out to assess mortality, morbidities, and the functional outcome of the care provided to them.

**Results:**

A total of 125 patients were studied (62 females). All of the patients were followed up for a mean duration of 6.28 years (range 1–23 years). The majority were located in the lumbosacral area. All of the patients underwent postnatal surgical intervention for MMC in Namazi Hospital. Mean age at surgery was 9.51 days. There were statistically significant differences between urinary and bowel incontinence and presence of scoliosis, MMT grading of the lower limbs, school attendance, number of readmissions, and requirement of laminectomy at the initial surgical intervention.

**Conclusions:**

This study is the first to characterize the long-term outcomes of MMC patients in Iran. This study illustrates that there is a great need for improved access to and coordination of care in antenatal, perioperative, and long-term stages to improve morbidity and mortality.

**Supplementary Information:**

The online version contains supplementary material available at 10.1186/s40001-024-01667-0.

## Introduction

Myelomeningocele (MMC) denotes a subset of neural tube defects (NTDs) that are caused by flawed closure of the fetal neural tube in the fourth week of pregnancy and result in neurological deficits of varying severity. Folic acid fortification during early pregnancy reduces the chance of NTDs occurrence [1]. However, because of the early closure of neural tube during fetal development, timely and easy access to prenatal care is critical in the early diagnosis of MMC and initiation of suitable intervention [2].

The severity of MMC depends on the location of the defect and extent of spinal cord exposure. NTDs symptoms can range from mild cosmetic abnormalities, such as dimples or tufts of hair on the lower back in spina bifida occulta cases, to urinary and bowel incontinence, hydrocephalus, severe paralysis, and chronic infections [2, 3]. To mitigate the complications of MMC, the standard of care in high-income countries in the initial phase comprises in utero diagnosis, cesarean section rather than vaginal birth, postnatal surgical closure of the open lesion within two days, and a rigorous follow-up aimed at detecting hydrocephalus or other complications, and treatment with surgical intervention as needed. The care for these patients should ideally be provided by multidisciplinary teams including several specialties (i.e., neurosurgery, urology, orthopedics, pediatrics, and physical therapists). One of the important accompanying conditions of MMC patients either present at birth or developing as the patient grows older, requiring multidisciplinary care is scoliosis, with 23% to 88% of MMC patients suffering from scoliosis. Scoliosis can result in respiratory compromise, reduced mobility, skin breakdown, posture issues, and exacerbation of neurological symptoms [4–6].

While the literate on MMC is quite rich and innovative in some aspects, there still remains considerable knowledge gaps regarding MMC. Long-term health outcomes, educational status, and transition to adulthood still require meticulous investigation in these patients. The need is exponentially more pronounced in among resource-limited settings where outcomes are generally inferior due to lack of coordinated care and delay in interventions and the patient population can be different from the mainstream literature from the developed countries [2, 7–11]. To the best of our knowledge, apart from the occasional case reports and small series, the literature regarding MMC in Iran and Middle east, more generally, are quite scares. In the present study, we have investigated the mortality and long-term outcome of MMC patients who were surgically treated at Namazi Hospital, the first study of this kind on Iranian patients.

## Materials and methods

### Patient population

This retrospective chart review study investigates children < 18 years old who underwent initial surgical intervention for congenital myelomeningocele between May 2001 and September 2020 in our institution, an academic tertiary referral center. Children with any forms of spina bifida other than myelomeningocele during the study period were excluded. Data extraction was performed for the following variables: age at surgery (days), gender, anatomic site of the lesion, preoperative cerebrospinal fluid leak, presence of lipid compartment in the defect, dural closure requiring synthetic graft, laminectomy, length of stay in the hospital at the initial admission, repeated admission, school attendance, urinary continence, bowel continence, ambulation dependency (Gross Motor Function Classification System; GMFCS), muscle strength of the lower limbs (Manual Muscle Testing; MMT), and mortality.

### Ethical approval and patient consent

The Ethics Committee of our institution waived ethical approval in view of the retrospective design of the study and the fact that all the interventions and follow-ups were part of the routine care. Written informed consent for participation and publication was collected from each participant at the time of operation.

### Statistical analysis

Interval data were reported as means with standard deviation (SD) while categorical data were reported as frequency and percentages. Interval data were analyzed using independent samples t-test and categorical data were analyzed using Chi-square or Fisher's exact test as appropriate. Statistical significance was set a priori at *P* < 0.05. All statistical analyses were carried out using IBM SPSS Statistics version 16 (IBM Corp., Armonk, N.Y., USA).

## Results

This study included 125 patients with an equal distribution of males and females. The mean age at surgery was 9.51 days and the majority of defect sites were at thoracolumbar and lumbar levels (86%). At the evaluation prior to surgery, 12% of patients had ruptured myelomeningoceles with CSF leaking. 62% of patients underwent laminectomy and 56% had autologous dura closure. The mean length of stay for surgical treatment was 10.96 days. At the last follow-up, 90% of patients were alive with a mean age of 6.35 years, while 10% had expired with a mean age at death of 5.62 years. 43% of patients were classified as GMFCS Level I and II and 27% of patients of school age had attended school. The majority of patients had urinary and bowel incontinence. Scoliosis was present in 35% of patients, with the majority being congenital. The overall mortality rate was 9.6% (Table 1).

**Table 1:** Characteristics of patients who underwent surgical closure of the MMC

Baseline characteristics	
Sex	
Male	63 (50%)
Female	62 (50%)
Mean age at surgery, days (SD)	9.51 (8.01)
Defect site	
Cervical	3 (2%)
Thoracic	3 (2%)
Thoracolumbar and lumbar	108 (86%)
Lumbosacral and sacral	11 (9%)
Rupture at the evaluation prior to operation (CSF leaking)	
Intact	110 (88%)
Ruptured	15 (12%)
Surgical treatment	
Mean length of stay, days (SD)	10.96 (9.83)
Laminectomy	
Performed	77 (62%)
Not performed	48 (38%)
Dura closure	
Autologous dura	69 (56%)
Synthetic dura	55 (44%)
Last follow-up	
Mortality	
Alive	113 (90%)
Mean age at last follow-up, years (SD)	6.35 (3.32)
Expired	12 (10%)
Mean age at death, years (SD)	5.62 (6.54)
Ambulation	
GMFCS Level I and II	54 (43%)
GMFCS Level III	26 (21%)
GMFCS Level IV	27 (22%)
GMFCS Level V	18 (14%)
Readmission	
No readmission	53 (42%)
One time	45 (36%)
Two times	16 (13%)
Three times	4 (3%)
Four or more times	6 (5%)
Education^a^	
Have not attended school	26 (58%)
Have attempted but failed	7 (16%)
Have attended school	12 (27%)
Urinary	
Continent	29 (23%)
Incontinent	96 (77%)
Fecal	
Continent	35 (28%)
Incontinent	90 (72%)
MMT grading	
0	28 (23%)
1	11 (9%)
2	9 (7%)
3	32 (26%)
4	27 (22%)
5	15 (10%)
Scoliosis	
Not present	80 (65%)
Congenital	38 (31%)
Acquired	6 (5%)
Mortality	12 (9.6%)

^a^Only includes the patients six years of age or older

The majority of deaths were attributable to sepsis and infections of urinary and respiratory systems (*n* = 7, 58%). Shunt malfunctions and unrelated traumas including traffic accidents were each responsible for the morality in two cases (17% each). Lastly, a patient was expired due a cardiac arrest without a specified cause.

The features that were significanlty or barly significantly associated with outcomes are summarized in Tables 2, 3. An extended version of the statistical analysis of all variables is available in Additional file 1: Tables S1-S4.

**Table 2:** The relationship between patient demographics, pre- and post-operational characteristics and mortality in children who underwent surgical treatment of myelomeningocele

MMC patients	GMFCS Level I and II (*n* = 54)	GMFCS Level III (*n* = 26)	GMFCS Level IV (*n* = 27)	GMFCS Level V (*n* = 18)	Total (%)	*p value*
Mean age at surgery, days (SD)	11.21 (7.326)	9.12 (7.384)	10.59 (6.326)	13.62 (20.425)	–	0.102^a^
Sex						**0.045**^b^
Female	24	17	9	12	62 (50%)	
Male	30	9	18	6	63 (50%)	
Dura closure						0.076^b^
Autologous dura	26	17	13	13	69 (56%)	
Synthetic dura	28	9	14	4	55 (44%)	
Readmission						0.064^b^
No readmission	25	12	10	6	53 (42%)	
One time	18	12	9	6	45 (36%)	
Two times	8	1	3	4	16 (13%)	
Three times	3	0	0	1	4 (3%)	
Four or more times	0	0	5	1	6 (5%)	
Urinary						**0.008**^b^
Continent	19	4	6	0	29 (23%)	
Incontinent	35	22	21	18	96 (77%)	
Bowel						**0.029**^b^
Continent	20	6	8	1	35 (28%)	
Incontinent	34	19	20	17	90 (72%)	
MMT grading						**0.000**^d^
0	3	2	11	12	28 (23%)	
1	2	5	2	2	11 (9%)	
2	0	4	3	2	9 (7%)	
3	14	9	8	1	32 (26%)	
4	21	4	1	1	27 (22%)	
5	14	1	0	0	15 (10%)	

A bold p-value indicates that the variable is significantly associated with the outcome

*MMC* myelomeningocele, *SD* standard deviation

^a^Student’s t-test,

^b^Fisher’s exact test

**Table 3:** The relationship between patient demographics, pre- and post-operational characteristics and ambulation dependence in children who underwent surgical treatment of myelomeningocele

MMC patients	Alive (*n* = 113)	Expired (*n* = 26)	Total (%)	*p value*
Length of stay, days (SD)	**9.91 (6.88)**	**20.5 (21.915)**	–	0.000^a^
Education^c^				**0.021**^b^
Not attended school	25	0	26 (58%)	
Have attempted but failed	5	2	7 (16%)	
Have attended school	12	0	12 (27%)	
Urinary				0.067^b^
Continent	29	0	29 (23%)	
Incontinent	84	12	96 (77%)	
Bowel				0.037^b^
Continent	35	0	35 (28%)	
Incontinent	78	12	90 (72%)	

A bold p-value indicates that the variable is significantly associated with the outcome

*MMC* myelomeningocele, *GMFCS* Gross Motor Function Classification System, *MMT* manual muscle testing, *SD* standard deviation

^a^Student’s t-test,

^b^Fisher’s exact test,

^c^Statistical analysis for school attendance only includes the patients six years of age or older

The analysis revealed a significant association between sex and GMFCS, with more males being classified in GMFCS levels III and IV, and more females in GMFCS levels I and II. Additionally, the type of dura closure used during surgery showed a marginal association with GMFCS levels, with autologous dura closure being more common in higher GMFCS levels. In terms of readmission rates, the analysis suggested an association with GMFCS levels, with higher GMFCS levels having a higher frequency of readmissions; however, the association failed to reached significance.

For urinary continence, there was a significant association with GMFCS levels, with a higher percentage of GMFCS Level V patients being incontinent compared to other levels. A similar association was observed for bowel continence, with almost all of the patients with the rate of incontinence dropping from roughly 60% in patients with GMFCS I and II to almost 100% in those with GMFCS V.

Table 2 also examines the outcomes for MMC patients in terms of survival. The length of stay was significantly shorter for patients who survived in the long-term compared to those who expired. Education status showed a significant association with survival, with only two of the patients who expired attempting school to attend school and fail. Regarding urinary and bowel continence, all of the patients who expired were incontinent.

There was no significant difference between the number of male and female patients in terms of bowel and urinary incontinence. The majority of patients in both the bowel and urinary incontinent groups were those who underwent laminectomy as a part of their initial treatment. The majority of patients in the bowel and urinary continent groups belonged to GMFCS Level I and II (43% and 43%, respectively). GMFCS Level III was the most common level in the bowel and urinary incontinent groups (21% and 21%, respectively) (Table 3).

There was significant correlation between urinary and bowel incontinence. Moreover, a significant difference was observed in the readmission rates between the bowel continent and incontinent groups. The bowel continent group had lower readmission rates, with 42% having no readmissions, compared to the bowel incontinent group, where more patients had readmission. A similar trend was observed between the urinary continent and incontinent groups (Table 4).

**Table 4:** The relationship between patient demographics, pre- and post-operational characteristics and urinary and bowel continence in children who underwent surgical treatment of myelomeningocele

MMC patients	Bowel continent (n = 37)	Bowel incontinent (n = 89)	Total (%)	*p value*
Sex				0.078^b^
Female	15	48	62 (50%)	
Male	22	41	63 (50%)	
Laminectomy				**0.002**^b^
Needed	30	47	77 (62%)	
Not needed	6	42	48 (38%)	
Ambulation				**0.029**^b^
GMFCS Level I and II	20	34	54 (43%)	
GMFCS Level III	6	20	26 (21%)	
GMFCS Level IV	8	19	27 (22%)	
GMFCS Level V	1	17	18 (14%)	
Readmission				**0.000**^b^
No readmission	22	31	53 (42%)	
One time	6	39	45 (36%)	
Two times	2	14	16 (13%)	
Three times	0	4	4 (3%)	
Four or more times	5	1	6 (5%)	
Education^c^				**0.000**^b^
Not attended school	20	6	26 (58%)	
Have attempted but failed	2	5	7 (16%)	
Have attended school	2	10	12 (27%)	
Urinary				**0.000**^b^
Continent	29	0	29 (23%)	
Incontinent	6	90	96 (77%)	
MMT grading				**0.000**^b^
0	1	27	28 (23%)	
1	4	7	11 (9%)	
2	4	5	9 (7%)	
3	3	29	32 (26%)	
4	14	13	27 (22%)	
5	7	8	15 (10%)	
Scoliosis				**0.000**^b^
Not present	31	49	80 (65%)	
Congenital	2	36	38 (31%)	
Acquired	3	3	6 (5%)	

MMC patients	Urinary continent (*n* = 29)	Urinary incontinent (*n* = 96)	Total (%)	*p value*
Sex				0.089^b^
Female	10	52	62 (50%)	
Male	19	44	63 (50%)	
Laminectomy				**0.009**^b^
Needed	24	53	77 (62%)	
Not needed	5	43	48 (38%)	
Ambulation				**0.013**^b^
GMFCS Level I and II	19	35	54 (43%)	
GMFCS Level III	4	22	26 (21%)	
GMFCS Level IV	6	21	27 (22%)	
GMFCS Level V	6	18	18 (14%)	
Readmission				**0.000**^b^
No readmission	21	32	53 (42%)	
One time	3	42	45 (36%)	
Two times	1	15	16 (13%)	
Three times	0	4	4 (3%)	
Four or more times	4	2	6 (5%)	
Education^c^				**0.043**^b^
Not attended school	15	11	26 (58%)	
Have attempted but failed	2	5	7 (16%)	
Have attended school	2	10	12 (27%)	
Bowel				**0.000**^b^
Continent	29	6	35 (28%)	
Incontinent	0	90	90 (72%)	
MMT grading				**0.000**^b^
0	1	27	28 (23%)	
1	3	8	11 (9%)	
2	4	5	9 (7%)	
3	0	32	32 (26%)	
4	12	15	27 (22%)	
5	7	8	15 (10%)	
Scoliosis				**0.002**^b^
Not present	26	54	80 (65%)	
Congenital	1	37	38 (31%)	
Acquired	2	4	6 (5%)	

*MMC* myelomeningocele, *GMFCS* Gross Motor Function Classification System, *MMT* manual muscle testing

^a^Student’s t-test

^b^Fisher’s exact test

^c^Statistical analysis for school attendance only includes the patients six years of age or older

A significant association was found between the presence of scoliosis and both bowel and urinary incontinence. Moreover, there was a significant association between MMT grading and bowel continence. Higher MMT was associated with bowel incontinence. Finally, there was a significant association between successfully attending school and urinary and bowel continence (*p* < 0.001). A higher percentage of patients who were urinary continent or bowel continent had attended school, while a higher percentage of patients who were urinary or bowel incontinent had either not attended school or were not successful in pursuing their education.

## Discussion

In this study, we reported the long-term follow-up of a cohort of infants who underwent postnatal surgical repair for MMC. The results of this study provide valuable insights into the long-term outcomes of MMC patients treated at a referral center in Southern Iran. The study included 125 patients, with an equal distribution of males and females. The mean age at surgery was 9.51 days, and the majority of MMC defects were located at the thoracolumbar and lumbar levels.

Prior to surgery, 12% of patients had ruptured myelomeningoceles with cerebrospinal fluid (CSF) leaking. The majority of patients underwent laminectomy (62%) and had autologous dura closure (56%). The mean length of stay for surgical treatment was 10.96 days. At the last follow-up, 90% of patients were alive, with a mean age of 6.35 years, while 10% had expired, with a mean age at death of 5.62 years.

In terms of functional outcomes, 43% of patients were classified as Gross Motor Function Classification System (GMFCS) Level I and II, indicating better motor function, while a significant portion (27%) of school-age patients had attended school. However, the majority of patients experienced urinary and bowel incontinence. Scoliosis was also prevalent in 35% of patients, with congenital scoliosis being the most common type observed. Scoliosis is one of the main spinal deformities that tends to accompany MMC. Patients with early-onset scoliosis are considered for orthotic treatment including braces. They can help patients sit comfortably as well as controlling further progression of the curve before the age of 10 [12]. In the management of scoliosis in patients with MMC, a holistic approach is essential. Patients with curves ranging from 10 to 25 degrees undergo regular surveillance through serial X-rays. For those with curves exceeding 25 degrees but falling below 40 to 45 degrees, bracing becomes a viable option as pointed out earlier, though questions persist regarding the efficacy of various types of braces. Surgical intervention is recommended for those with curves surpassing 40 to 45 degrees, with surgical fusion being the primary method. However, the decision for surgery should be approached cautiously, considering the substantial risks outlined in the literature. Alternative treatments such as physical therapy, electrical stimulation, nutrition, and spinal manipulation have yet to demonstrate efficacy in scoliosis management. Recognizing the need for personalized care, operative decisions must carefully weigh patient-specific factors, skeletal maturity, deformity progression, socioeconomic considerations, and the experience of the surgical team [6, 12, 13].

The care for patients with MMC should start prenatally. The neural tube closes by week four of fetal development. As a result, the diagnosis should not be delayed [7, 14]. Early prenatal diagnosis of MMC by ultrasonography provide the parents with the option of timely termination of pregnancy [2]. In the current convention of MMC treatment, baring the termination of pregnancy, two options are available: postnatal open and prenatal MMC repair. The initial step in the surgical treatment of MMC is MRI or CT scanning to define and fully characterize the lesion [15]. Fetal MMC surgery was designed to improve children's development and long-term quality of life; however, it is associated with maternal and fetal risks and complications [16, 17]. There is a lack of clinical or educational infrastructure in many developing countries for the prevention, early detection, and/or promotion of the care of MMC patients. It is combined with the hesitancy toward the prenatal care and timely termination of pregnancy in certain population and the poverty in others. The average age at surgery in this study was 9.51 days, which differs from previous reports of four days in Southwest Nigeria [18], 21 days in Zambia [11], and six days in Uganda [8] for developing countries. In contrast, developed settings tend to repair the defect earlier, with reports of under two days in the MOMS study [19] and another report from North Carolina [20] while the Rotterdam experience found that 79% of their patients were operated on after two days of age [9]. The average post-operative length of stay in this study was 11 days. Previous studies have reported a mean length of stay of three days in Zambia [11], 20 days in Uganda [8], and 11.9 in the nationwide inpatient sample myelomeningocele [21].

26% of the patients had GMFCS Level IV and V, which is comparable to the 21% independently walking patients in the postnatal group of the MOMS trial and the rate in other reports [9, 22]. However, superior outcome was reported in a small series from Lurie Children’s Hospital with roughly 10% of the patients unable to ambulate in the community [23].

School attendance and the mortality rate we reported in this study are considerably inferior to the reports from developed countries and similar to the developing countries [8, 9, 24] [8]. Mortality rate was 4% in either arm of the MOMs trial and 3% in UPMC cohort at 30 months and about 2 percent in the Rotterdam experience while it was close to 20% in long-term follow-up report from Uganda [22, 25].

The damage to the nerve roots of the spinal cord causes urinary or bowel incontinence, one of the major complications of MMC. Krogh et al. reported that 66% of the patients with myelomeningocele over the age of 6 who suffered from bowel incontinence stated that their social activities or quality of life was influenced partly or majorly by this problem [26]. Verhoef et al. reported that 69.7% and 77.0% of patients with urinary and bowel incontinence, respectively, perceived incontinence as a problem [27]. Lie et al. reported that the majority of the patients suffering from urinary incontinence considered it a stress factor. Van Gool et al. reported that patients experiencing improvements in urinary function considered it an improvement, giving them additional independence. Therefore, improving the urinary function is therefore an important goal of multidisciplinary and specialized medical care [28, 29].

The rate of urinary and bowel incontinence reported here (roughly 70%) is comparable to other studies such as the National Spina Bifida Patient Registry (roughly 60%) [27, 30]. Nevertheless, similar to the other outcomes in some instances where drastically superior results were achieved such as a rate of close to 20% incontinence in patients reported by Behbahani et al. from Lurie Children’s Hospital [23]. As outlined in our results and reported previously, there is a correlation between the urinary and bowel continence and school attendance [31]. This finding may be emphasized in settings with limited special education facilities, like the present study.

Generally, the outcome of the patients presented in the present study is inferior to the ones reported from developed countries and more in line with the ones from developing countries. The patients who expired in the long-term follow-up were exclusively from the patient group who had urinary or bowel incontinence. On top of that, the education of the patients was correlated with their urinary and bowel function.

In our setting, the management of MMC is complicated by the hesitancy toward the prenatal care and timely termination of pregnancy in certain population and the poverty and poor access to timely healthcare in others. Limited resources, including funding, healthcare infrastructure, and skilled healthcare professionals, pose significant barriers to the provision of appropriate long-term care. Inadequate availability of neurosurgical expertise contributes to delayed or missed follow-up visits, leading to suboptimal patient outcomes. Furthermore, geographical barriers, including long distances between patients' homes and healthcare centers, lack of transportation infrastructure, and limited accessibility to specialized care facilities, hinder regular follow-up. Socioeconomic factors, such as poverty and low health literacy among patients and caregivers, can also impact the compliance and understanding of the importance of follow-up visits [16].

There is evidence that community-based support and follow-up for MMC patients and their parents significantly promote patient survival following surgery [32]. Although published data remain limited, a higher incidence of MMC and inferior long-term outcomes in certain aspects in Iran can be justified according to the aforementioned factors. Poor record keeping and follow-up system makes managing the long-term complications of MMC patients difficult. A well-structured and regular follow-up is essential to monitor the patient's neurological development, identify potential complications, and provide appropriate interventions. However, developing countries often face numerous challenges in ensuring a comprehensive follow-up for these patients. Additionally, follow-up visits are essential in addressing other associated conditions such as hydrocephalus, tethered cord syndrome, Chiari malformation, etc., ensuring timely intervention and preventing potential long-term disabilities.

This report provides information for surgeons and parents about the long-term outcome, complications and risks of postnatal MMC repair. Moreover, it allows comparison to long-term outcomes in the more developed and resourceful settings. Furthermore, with the standard treatment for MMC transitioning toward prenatal surgery, the present report provides a historical cohort to evaluate against future antenatal-treated MMC series.

## Limitations

Our study is limited by its retrospective design and the inevitable loss of data. For instance, the World Health Organization has chosen Namazi Hospital as a center of referral for surgical correction of MMC. However, due to limited access, we had to exclude patients with other nationalities due to limitations in availability of their data in our registry and lack of follow-up.

## Conclusion

This study provides a summary of the outcomes for infants with MMC managed at our center. The results of this study provide valuable insights into the long-term outcomes of MMC patients treated at a referral center in Southern Iran. We showed that children with urinary incontinence, failure to attend school, and longer hospital stay were at a higher risk for mortality during their follow-up. Moreover, urinary and bowel incontinence were associated with more readmissions, lower GMFCS, MMT, and presence of scoliosis. We believe that there is a great need for follow-up of MMC patients both until and beyond 18 years of age into adulthood. The findings of this study can contribute to our understanding of MMC outcomes and inform clinical management and interventions for patients with this condition.

### Supplementary Information

**Additional file 1: Table S1.** The relationship between patient demographics, pre- and post-operational characteristics and Gross Motor Function Classification System (GMFCS) in children undergoing surgical treatment of myelomeningocele. **Table S2.** The relationship between patient demographics, pre- and post-operational characteristics and mortality in children undergoing surgical treatment of myelomeningocele. **Table S3.** The relationship between patient demographics, pre- and post-operational characteristics and fecal continence in children undergoing surgical treatment of myelomeningocele. **Table S4.** The relationship between patient demographics, pre- and post-operational characteristics and mortality in children undergoing surgical treatment of myelomeningocele.

## Data Availability

All data generated or analyzed during this study are included in the final published article.
