# Supplementary material for: 2- to 20-year myelomeningocele follow-up outcomes from a referral center in Southern Iran: the Shiraz experience
Source: Eur J Med Res. 2024 Mar 25;29:200. doi: 10.1186/s40001-024-01667-0 (PMC10962193; doi:10.1186/s40001-024-01667-0)
Supplement: Supplementary file 1 — Additional file 1: Table S1. The relationship between patient demographics, pre- and post-operational characteristics and Gross Motor Function Classification System (GMFCS) in children undergoing surgical treatment of myelomeningocele. Table S2. The relationship between patient demographics, pre- and post-operational characteristics and mortality in children undergoing surgical treatment of myelomeningocele. Table S3. The relationship between patient demographics, pre- and post-operational characteristics and fecal continence in children undergoing surgical treatment of myelomeningocele. Table S4. The relationship between patient demographics, pre- and post-operational characteristics and mortality in children undergoing surgical treatment of myelomeningocele. [file 40001_2024_1667_MOESM1_ESM.docx]

| MMC patients | GMFCS Level I and II (n= 54) | GMFCS Level III (n= 26) | GMFCS Level IV (n= 27) | GMFCS Level V (n= 18) | Total (%) | *p value* |
| --- | --- | --- | --- | --- | --- | --- |
| Mean age at surgery, days (SD) | 11.21 (7.326) | 9.12 (7.384) | 10.59 (6.326) | 13.62 (20.425) | - | .102^a^ |
| Length of stay, days (SD) | 8.48 (6.074) | 8.40 (7.990) | 9.89 (9.133) | 13.61 (10.399) | - | .553^a^ |
| Sex |  |  |  |  |  | **.045**^b^ |
| Female | 24 | 17 | 9 | 12 | 62 (50%) |  |
| Male | 30 | 9 | 18 | 6 | 63 (50%) |  |
| Rupture at the the Evaluation Prior to Operation (CSF leaking) |  |  |  |  |  | .222^b^ |
| Intact | 46 | 24 | 26 | 14 | 110 (88%) |  |
| Ruptured | 8 | 2 | 1 | 4 | 15 (12%) |  |
| Site of lesion |  |  |  |  |  | .657^b^ |
| Cervical | 1 | 0 | 1 | 1 | 3 (2%) |  |
| Thoracic | 3 | 0 | 0 | 0 | 3 (2%) |  |
| Thoracolumbar and Lumbar | 45 | 23 | 23 | 17 | 108 (86%) |  |
| Lumbosacral and Sacral | 5 | 3 | 3 | 0 | 11 (9%) |  |
| Laminectomy |  |  |  |  |  | .581^b^ |
| Needed | 32 | 19 | 16 | 10 | 77 (62%) |  |
| Not needed | 22 | 7 | 11 | 8 | 48 (38%) |  |
| Dura closure |  |  |  |  |  | .076^b^ |
| Autologous dura | 26 | 17 | 13 | 13 | 69 (56%) |  |
| Synthetic dura | 28 | 9 | 14 | 4 | 55 (44%) |  |
| Readmission |  |  |  |  |  | .064^b^ |
| No Readmission | 25 | 12 | 10 | 6 | 53 (42%) |  |
| One Time | 18 | 12 | 9 | 6 | 45 (36%) |  |
| Two Times | 8 | 1 | 3 | 4 | 16 (13%) |  |
| Three Times | 3 | 0 | 0 | 1 | 4 (3%) |  |
| Four or more Times | 0 | 0 | 5 | 1 | 6 (5%) |  |
| Education^c^ |  |  |  |  |  | 0.337^b^ |
| Not Attended School | 15 | 6 | 3 | 2 | 26 (58%) |  |
| Have Attempted but Failed | 2 | 3 | 2 | 0 | 7 (16%) |  |
| Have Attended School | 4 | 2 | 4 | 2 | 12 (27%) |  |
| Urinary |  |  |  |  |  | **.008**^b^ |
| Continent | 19 | 4 | 6 | 0 | 29 (23%) |  |
| Incontinent | 35 | 22 | 21 | 18 | 96 (77%) |  |
| Fecal |  |  |  |  |  | **.029**^b^ |
| Continent | 20 | 6 | 8 | 1 | 35 (28%) |  |
| Incontinent | 34 | 19 | 20 | 17 | 90 (72%) |  |
| MMT Grading |  |  |  |  |  | **.000**^d^ |
| 0 | 3 | 2 | 11 | 12 | 28 (23%) |  |
| 1 | 2 | 5 | 2 | 2 | 11 (9%) |  |
| 2 | 0 | 4 | 3 | 2 | 9 (7%) |  |
| 3 | 14 | 9 | 8 | 1 | 32 (26%) |  |
| 4 | 21 | 4 | 1 | 1 | 27 (22%) |  |
| 5 | 14 | 1 | 0 | 0 | 15 (10%) |  |
| Scoliosis |  |  |  |  |  | .133^b^ |
| Not Present | 38 | 18 | 12 | 12 | 80 (65%) |  |
| Congenital | 14 | 7 | 11 | 6 | 38 (31%) |  |
| Acquired | 2 | 0 | 4 | 0 | 6 (5%) |  |

Table S1. The relationship between patient demographics, pre- and post-operational characteristics and Gross Motor Function Classification System (GMFCS) in children undergoing surgical treatment of myelomeningocele. MMC; Myelomeningocele, SD; Standard Deviation, CSF; Cerebrospinal Fluid. ^a^ ANOVA, ^b^ Fisher’s Exact Test, ^c^ Statistical analysis for school attendance only includes the patients reaching six years of age or older, ^d^ Chi-square

| MMC patients | Alive (n=113) | Expired (n=12) | Total (%) | *p value* |
| --- | --- | --- | --- | --- |

| Mean age at surgery, days (SD) | 9.55 (8.23) | 5.728 (8.43) | - | 0.848^a^ |
| --- | --- | --- | --- | --- |
| Length of stay, days (SD) | 9.91 (6.88) | 20.5 (21.915) | - | **0.000**^a^ |
| Sex |  |  |  | .763^b^ |
| Female | 57 | 5 | 62 (50%) |  |
| Male | 56 | 7 | 63 (50%) |  |
| Rupture at the evaluation prior to operation (CSF leaking) |  |  |  | .158^b^ |
| Intact | 101 | 9 | 110 (88%) |  |
| Ruptured | 12 | 3 | 15 (12%) |  |
| Site of lesion |  |  |  | .243^b^ |
| Cervical | 3 | 0 | 3 (2%) |  |
| Thoracic | 3 | 0 | 3 (2%) |  |
| Thoracolumbar and Lumbar | 99 | 9 | 108 (86%) |  |
| Lumbosacral and Sacral | 8 | 3 | 11 (9%) |  |
| Laminectomy |  |  |  | .766^b^ |
| Needed | 69 | 8 | 77 (62%) |  |
| Not needed | 44 | 4 | 48 (38%) |  |
| Dura closure |  |  |  | 1.000^b^ |
| Autologous dura | 62 | 7 | 69 (56%) |  |
| Synthetic dura | 50 | 5 | 55 (44%) |  |
| Ambulation |  |  |  | .609^b^ |
| GMFCS Level I and II | 50 | 4 | 54 (43%) |  |
| GMFCS Level III | 23 | 3 | 26 (21%) |  |
| GMFCS Level IV | 25 | 2 | 27 (22%) |  |
| GMFCS Level V | 15 | 3 | 18 (14%) |  |
| Readmission |  |  |  | .383^b^ |
| No Readmission | 50 | 3 | 53 (42%) |  |
| One Time | 40 | 5 | 45 (36%) |  |
| Two Times | 13 | 3 | 16 (13%) |  |
| Three Times | 4 | 0 | 4 (3%) |  |
| Four or more Times | 5 | 1 | 6 (5%) |  |
| Education^c^ |  |  |  | **0.021**^b^ |
| Not Attended School | 25 | 0 | 26 (58%) |  |
| Have Attempted but Failed | 5 | 2 | 7 (16%) |  |
| Have Attended School | 12 | 0 | 12 (27%) |  |
| Urinary |  |  |  | .067^b^ |
| Continent | 29 | 0 | 29 (23%) |  |
| Incontinent | 84 | 12 | 96 (77%) |  |
| Fecal |  |  |  | **.037**^b^ |
| Continent | 35 | 0 | 35 (28%) |  |
| Incontinent | 78 | 12 | 90 (72%) |  |
| MMT Grading |  |  |  | .320^b^ |
| 0 | 24 | 4 | 28 (23%) |  |
| 1 | 8 | 3 | 11 (9%) |  |
| 2 | 8 | 1 | 9 (7%) |  |
| 3 | 30 | 2 | 32 (26%) |  |
| 4 | 25 | 2 | 27 (22%) |  |
| 5 | 15 | 0 | 15 (10%) |  |
| Scoliosis |  |  |  | 1.000^b^ |
| Not Present | 72 | 8 | 80 (65%) |  |
| Congenital | 34 | 4 | 38 (31%) |  |
| Acquired | 6 | 0 | 6 (5%) |  |

Table S2. The relationship between patient demographics, pre- and post-operational characteristics and mortality in children undergoing surgical treatment of myelomeningocele. MMC; Myelomeningocele, GMFCS; Gross Motor Function Classification System, MMT; Manual Muscle Testing, SD; Standard Deviation, CSF; Cerebrospinal Fluid. ^a^ Student's t-test, ^b^ Fisher’s Exact Test, ^c^ Statistical analysis for school attendance only includes the patients reaching six years of age or older.

| MMC patients | Fecal Continent (n=37) | Fecal Incontinent (n=89) | Total (%) | *p value* |
| --- | --- | --- | --- | --- |

| Mean age at surgery, days (SD) | 8.84 (8.25) | 9.71 (7.97) | - | 0.409^a^ |
| --- | --- | --- | --- | --- |
| Length of stay, days (SD) | 9.61 (5.13) | 11.37 (10.85) | - | 0.613^a^ |
| Sex |  |  |  | .078^b^ |
| Female | 15 | 48 | 62 (50%) |  |
| Male | 22 | 41 | 63 (50%) |  |
| Rupture at the evaluation prior to operation (CSF leaking) |  |  |  | .653^b^ |
| Intact | 32 | 78 | 110 (88%) |  |
| Ruptured | 3 | 12 | 15 (12%) |  |
| Site of lesion |  |  |  | .197^b^ |
| Cervical | 1 | 2 | 3 (2%) |  |
| Thoracic | 0 | 3 | 3 (2%) |  |
| Thoracolumbar and Lumbar | 35 | 73 | 108 (86%) |  |
| Lumbosacral and Sacral | 0 | 11 | 11 (9%) |  |
| Laminectomy |  |  |  | **.002**^b^ |
| Needed | 30 | 47 | 77 (62%) |  |
| Not needed | 6 | 42 | 48 (38%) |  |
| Dura closure |  |  |  | .695^b^ |
| Autologous dura | 21 | 48 | 69 (56%) |  |
| Synthetic dura | 15 | 40 | 55 (44%) |  |
| Ambulation |  |  |  | **.029**^b^ |
| GMFCS Level I and II | 20 | 34 | 54 (43%) |  |
| GMFCS Level III | 6 | 20 | 26 (21%) |  |
| GMFCS Level IV | 8 | 19 | 27 (22%) |  |
| GMFCS Level V | 1 | 17 | 18 (14%) |  |
| Readmission |  |  |  | **.000**^b^ |
| No Readmission | 22 | 31 | 53 (42%) |  |
| One Time | 6 | 39 | 45 (36%) |  |
| Two Times | 2 | 14 | 16 (13%) |  |
| Three Times | 0 | 4 | 4 (3%) |  |
| Four or more Times | 5 | 1 | 6 (5%) |  |
| Education^c^ |  |  |  | **.000**^b^ |
| Not Attended School | 20 | 6 | 26 (58%) |  |
| Have Attempted but Failed | 2 | 5 | 7 (16%) |  |
| Have Attended School | 2 | 10 | 12 (27%) |  |
| Urinary |  |  |  | **.000**^b^ |
| Continent | 29 | 0 | 29 (23%) |  |
| Incontinent | 6 | 90 | 96 (77%) |  |
| MMT Grading |  |  |  | **.000**^b^ |
| 0 | 1 | 27 | 28 (23%) |  |
| 1 | 4 | 7 | 11 (9%) |  |
| 2 | 4 | 5 | 9 (7%) |  |
| 3 | 3 | 29 | 32 (26%) |  |
| 4 | 14 | 13 | 27 (22%) |  |
| 5 | 7 | 8 | 15 (10%) |  |
| Scoliosis |  |  |  | **.000**^b^ |
| Not Present | 31 | 49 | 80 (65%) |  |
| Congenital | 2 | 36 | 38 (31%) |  |
| Acquired | 3 | 3 | 6 (5%) |  |

Table S3. The relationship between patient demographics, pre- and post-operational characteristics and fecal continence in children undergoing surgical treatment of myelomeningocele. MMC; Myelomeningocele, GMFCS; Gross Motor Function Classification System, MMT; Manual Muscle Testing, SD; Standard Deviation, CSF; Cerebrospinal Fluid. ^a^ Student's t-test, ^b^ Fisher’s Exact Test, ^c^ Statistical analysis for school attendance only includes the patients reaching six years of age or older.

| MMC patients | Urinary Continent (n=29) | Urinary Incontinent (n=96) | Total (%) | *p value* |
| --- | --- | --- | --- | --- |

| Mean age at surgery, days (SD) | 8.5 (9.16) | 9.87 (8.30) | - | 0.511^a^ |
| --- | --- | --- | --- | --- |
| Length of stay, days (SD) | 9.61 (5.41) | 11.71 (11.81) | - | 0.466^a^ |
| Sex |  |  |  | .089^b^ |
| Female | 10 | 52 | 62 (50%) |  |
| Male | 19 | 44 | 63 (50%) |  |
| Rupture at the evaluation prior to operation (CSF leaking) |  |  |  | 1.000^b^ |
| Intact | 26 | 84 | 110 (88%) |  |
| Ruptured | 3 | 12 | 15 (12%) |  |
| Site of lesion |  |  |  | .168^b^ |
| Cervical | 1 | 2 | 3 (2%) |  |
| Thoracic | 0 | 3 | 3 (2%) |  |
| Thoracolumbar and Lumbar | 28 | 80 | 108 (86%) |  |
| Lumbosacral and Sacral | 0 | 11 | 11 (9%) |  |
| Laminectomy |  |  |  | **.009**^b^ |
| Needed | 24 | 53 | 77 (62%) |  |
| Not needed | 5 | 43 | 48 (38%) |  |
| Dura closure |  |  |  | .871^b^ |
| Autologous dura | 17 | 52 | 69 (56%) |  |
| Synthetic dura | 12 | 43 | 55 (44%) |  |
| Ambulation |  |  |  | **.013**^b^ |
| GMFCS Level I and II | 19 | 35 | 54 (43%) |  |
| GMFCS Level III | 4 | 22 | 26 (21%) |  |
| GMFCS Level IV | 6 | 21 | 27 (22%) |  |
| GMFCS Level V | 6 | 18 | 18 (14%) |  |
| Readmission |  |  |  | **.000**^b^ |
| No Readmission | 21 | 32 | 53 (42%) |  |
| One Time | 3 | 42 | 45 (36%) |  |
| Two Times | 1 | 15 | 16 (13%) |  |
| Three Times | 0 | 4 | 4 (3%) |  |
| Four or more Times | 4 | 2 | 6 (5%) |  |
| Education^c^ |  |  |  | **0.043**^b^ |
| Not Attended School | 15 | 11 | 26 (58%) |  |
| Have Attempted but Failed | 2 | 5 | 7 (16%) |  |
| Have Attended School | 2 | 10 | 12 (27%) |  |
| Fecal |  |  |  | **.000**^b^ |
| Continent | 29 | 6 | 35 (28%) |  |
| Incontinent | 90 | 0 | 90 (72%) |  |
| MMT Grading |  |  |  | **.000**^b^ |
| 0 | 1 | 27 | 28 (23%) |  |
| 1 | 3 | 8 | 11 (9%) |  |
| 2 | 4 | 5 | 9 (7%) |  |
| 3 | 0 | 32 | 32 (26%) |  |
| 4 | 12 | 15 | 27 (22%) |  |
| 5 | 7 | 8 | 15 (10%) |  |
| Scoliosis |  |  |  | **.002**^b^ |
| Not Present | 26 | 54 | 80 (65%) |  |
| Congenital | 1 | 37 | 38 (31%) |  |
| Acquired | 2 | 4 | 6 (5%) |  |

Table S4. The relationship between patient demographics, pre- and post-operational characteristics and mortality in children undergoing surgical treatment of myelomeningocele. MMC; Myelomeningocele, GMFCS; Gross Motor Function Classification System, MMT; Manual Muscle Testing, SD; Standard Deviation, CSF; Cerebrospinal Fluid. ^a^ Student's t-test, ^b^ Fisher’s Exact Test, ^c^ Statistical analysis for school attendance only includes the patients reaching six years of age or older.
